# Comprehensive tumor profiling identifies numerous biomarkers of drug response in cancers of unknown primary site: Analysis of 1806 cases

**DOI:** 10.18632/oncotarget.2574

**Published:** 2014-10-31

**Authors:** Zoran Gatalica, Sherri Z. Millis, Semir Vranic, Ryan Bender, Gargi D. Basu, Andreas Voss, Daniel D. Von Hoff

**Affiliations:** ^1^ Caris Life Sciences, Phoenix, United States of America; ^2^ Department of Pathology, Clinical Center, University of Sarajevo, Sarajevo, Bosnia and Herzegovina; ^3^ Translational Genomic Research Institute and Virginia G Piper Cancer Center, Phoenix, United States of America

## Abstract

**Background:**

Cancer of unknown primary (CUP) accounts for approximately 3% of all malignancies. Despite extensive laboratory and imaging efforts, the primary site usually cannot be unequivocally confirmed, and the treatment for the most part remains empirical. Recently, identification of common cancer pathway alterations in diverse cancer lineages has offered an opportunity to provide targeted therapies for patients with CUP, irrespective of the primary site.

**Patients and Methods:**

1806 cancers of unknown primary were identified among more than 63,000 cases profiled at Caris Life Sciences. Multiplatform profiling of the tumor samples included immunohistochemistry, gene sequencing and *in situ* hybridization methods in an effort to identify changes in biomarkers that are predictive of drug responses.

**Results:**

Biomarkers associated with a potential drug benefit were identified in 96% of cases. Biomarkers identified included those associated with potential benefit in nearly all classes of approved cancer drugs (cytotoxic, hormonal, targeted biological drugs). Additionally, biomarkers associated with a potential lack of benefit were identified in numerous cases, which could further refine the management of patients with CUP.

**Conclusion:**

Comprehensive biomarker profiling of CUP may provide additional choices in treatment of patients with these difficult to treat malignancies.

## INTRODUCTION

Cancer of unknown primary (CUP) is a heterogeneous clinicopathologic syndrome constituting 3% of all malignancies [1]. Historically, CUP was associated with a poor prognosis [1–4], and patients are offered limited, non-selective (“broad-spectrum”) treatment choices [1, 2, 4–6]. Extensive tumor sample investigations to identify the presumed tissue of origin had been developed, utilizing gene expression arrays and immunohistochemistry [3, 4, 7], which may provide an indication of the potential primary site [8]. However, in true CUP by definition, the diagnosis of the primary cancer cannot be verified. In all such cases administration of presumed primary site-specific therapy remains empirical and for most cases is not driven by predictive biomarkers [5]. Recently, identification of shared, actionable pathway alterations in tumors from diverse primary sites has offered an opportunity to suggest pathway-specific therapies independent of tissue lineage [9–12]. Therefore, one of the most relevant strategies for effective use of targeted treatment modalities in CUP is the proper identification of the predictive biomarkers, and use of individualized therapies driven by those identified aberrations [13].

In the present study we reviewed 1806 CUP case data obtained through multi-platform tumor profiling at a single reference laboratory, and successfully identified numerous predictive biomarkers which could lead to improvement in management of CUP.

## RESULTS

### Patients' and tumor sample characteristics

The cohort included 44% male and 56% female patients with respective mean ages of 61.8 and 63.0 years (range, 1 to 92) with only 7 patients (~0.4%) under the age of 20 years. The most common biopsy site was liver (24%), followed by lymph nodes (17%), skin/soft tissues (14%), lung (8%), alimentary tract (7%), bones/joints (6%), and a variety of other sites at less than 5% each.

The diagnosis of CUP was previously established in all cases from the referring clinicians and institutions; IHC stains performed at the referring pathology laboratories did not unequivocally establish the primary site in any of the cases tested. Upon accessioning, all cases were reviewed by a board certified pathologist to verify the adequacy of the sample. The majority of the cases (82%) were confirmed to be carcinomas, of which 45% were adenocarcinomas. The remaining 18% were neuroendocrine tumors (9%), undifferentiated malignancies (8%) and sarcomas and melanomas (~1%).

### Biomarker expression and drug associations

**Figure 1:**
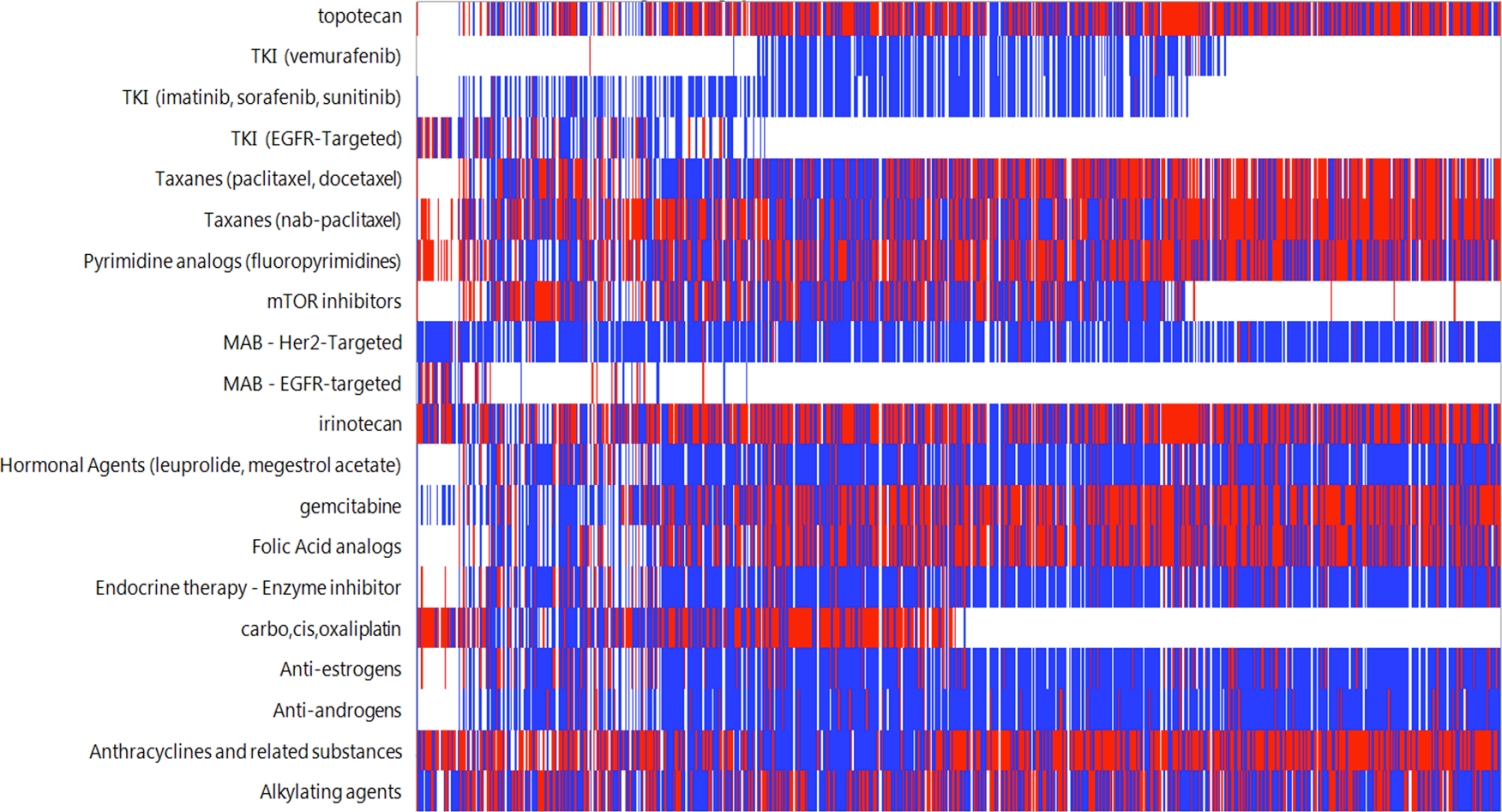
Drug associations based on molecular aberrations found in CUP cases White indicates no association, blue indicates lack of benefit association, and red indicates benefit association to a therapy Each column represents an individual patient.

In all but 15 cases (<1%) the identified biomarker aberration resulted in an association with either a benefit or lack of benefit with known therapeutics. In 96% of cases, a biomarker was found that identified a therapy of potential benefit. In 98% of cases, a biomarker was found that identified a therapy with potential lack of benefit. The frequencies of associations are shown in Figure 1.

### Protein expression

**Figure 2:**
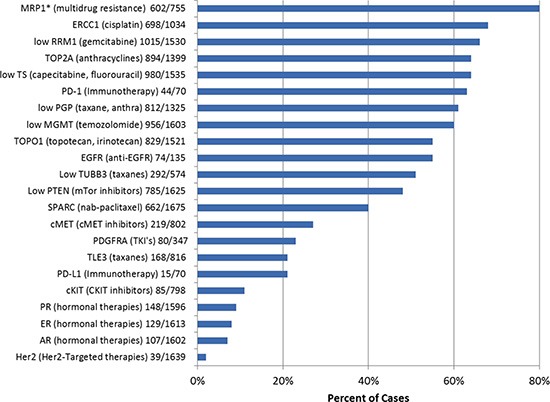
Protein biomarkers identified by immunohistochemistry, including potential association with therapies.* indicates test no longer offered

A number of cytotoxic and targeted therapy responses are dependent on either the presence or overexpression of a protein or loss of or reduced expression of a protein. IHC was utilized to assess levels of protein expression in the specimens. All IHC stains were reviewed by a board certified pathologist and interpreted utilizing thresholds published in the literature. IHC profiling of the protein biomarkers and their drug associations are outlined in Figure 2.

The most commonly identified protein changes associated with a potential benefit using targeted therapies were over expression of two topoisomerases (Topo1 and Topo2α), identified in 55% and 64% of all CUPs, respectively. Overexpression of targetable steroid receptors (estrogen receptor, 8%, progesterone receptor, 9%, androgen receptor, 7%) was seen in 20% of all CUP cases (4% of cases had co-expression of at least 2 steroid receptors). Loss of the expression of MGMT [O-6-methylguanine-DNA methyltransferase] was found in 40% of CUPs. Decreased expression or loss of PTEN [phosphatase and tensin homolog] was identified in 52% of cases, while under-expression of TS [Thymidylate Synthase] was recorded in 64% of the cases.

Protein expression changes that were associated with a lack of potential benefit to therapeutic agents were also investigated. The most common changes in this group were overexpression of MRP1 [multidrug resistance–associated protein 1] detected in 80% of cases, and overexpression of BCRP [breast cancer resistance protein, a member of the superfamily of ABC transporter proteins] in 75% of cases. ERCC1 [excision repair cross-complementation group 1] was overexpressed in 32% of the CUP cases, suggesting potential resistance to platinum-based chemotherapies.

Programmed cell death receptor (PD-1) and its ligand (PD-L1), which can be targeted by monoclonal antibodies (Anti-PD-1 antibodies: Nivolumab, MK-3475, and pidilizumab), were studied in 70 recently analyzed CUP cases. PD-1 expression in tumor-infiltrating lymphocytes and PD-L1 cancer cells' expression were observed in 63% and 21% of the cases, respectively. Co-expression of both markers was detected in 11 out of 70 (16%) tested cases.

### Gene alterations (copy number variations, re-arrangements and mutations)

The most commonly amplified genes in our series were *EGFR* and *HER2* (17% and 5% of the cases, respectively). *cMET* and *TOP2A* were rarely amplified (1% and 3%, respectively), despite the common expression of the respective proteins (Table 1 and Figure 1). Translocations of *ALK* and *ROS1* were not observed in any cases analyzed (108 and 12 cases, respectively).

**Table 1:** Gene copy number/translocation analysis (FISH and/or CISH)

Gene	Amplification rate (%)	Drug (class) associations
***cMET***	8/577 (1%)	cMET TKI's
***EGFR***	83/490 (17%)	Anti-EGFR antibodies, TKI's
***HER2***	42/879 (5%)	Anti-HER2 antibodies, TKI's
***PIK3CA***	1/7 (14%)	PAM pathway inhibitors
***TOP2A***	4/151 (3%)	Anthracyclines
***ALK***	0/108 (0%)	Ceritinibx (TKI)
***ROS1***	0/12 (0%)	Crizotinib (TKI)

PAM - the phosphoinositide 3-kinase-Akt-mammalian target of rapamycin pathway

TKI – Tyrosine kinase inhibitors

**Figure 3:**
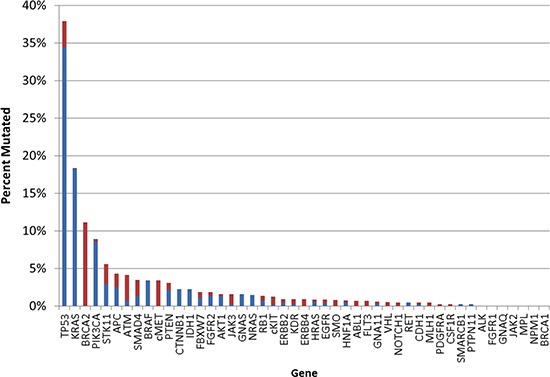
Gene mutation (n=47) frequency in CUP Blue indicates pathogenic mutation; red indicates variant of unknown significance

The most commonly mutated genes in this CUP cohort were *TP53* and *KRAS* (38% and 18%, respectively) with three additional genes that were mutated with a frequency at ≥5% (*BRCA2, PIK3CA,* and *STK11*) (Figure 3). Although overexpression of EGFR protein was common (detected in 55% of the cases), *EGFR* mutations were identified in only 4 out of 473 CUPs tested (E746_A750del, L858R, and two with M600T). A case of activating *EGFR* mutation (E746_A750del) was identified in the biopsy of a young woman's sacrum (without radiologic evidence of lung or other primary cancer and equivocal IHC profile, which included diverse possible primary sites such as upper GI tract, pancreas and biliary tree, as well as triple-negative breast cancer). She was treated with erlotinib and had a near-complete clinical response at 5 months.

Similarly, cKit protein expression was observed in 11% of the cases, while *cKIT* mutations were found in only 1% of cases (7 of 559). Six out of 7 detected *cKIT* mutations represented variants of unknown significance. However, pathogenic *cKIT* mutation (P551_M552del) in a neuroendocrine carcinoma of unknown primary was associated with over-expression of the protein and the patient received targeted therapy (Imatinib), which resulted in durable (>46 months) response [14].

Analysis of the *BRAF* mutated cases (n=24) showed that 20 cases (83%) were carcinomas (all positive for pan-cytokeratin and negative for melanocytic markers [e.g. S-100, Melan-A and HMB-45] by IHC; 5 were suggestive of colorectal primary with CK20 and CDX-2 positivity) while 4 cases (17%) were non-epithelial CUPs (all negative for pan-cytokeratins: three undifferentiated malignancies and one malignant melanoma). Evidence of a potential role of EGFR pathway activation (EGFR protein over expression or gene mutation) was seen in 10/19 of these cases (53%); *KRAS* mutations were identified in three cases (13%; Table 2). Of note, EGFR activation and co-mutation with *KRAS* favored tumors with non-V600 *BRAF* mutations. EGFR overexpression was present in 6/9 non-V600 mutated samples while 3/10 V600 mutated samples had EGFR overexpression. All three samples with *KRAS* co-mutation had non-V600 mutations. This suggests that non-V600 *BRAF* mutations may be sufficient to initiate cancer but may require further alterations in the RAS pathway for tumor progression, as non-V600 mutations are not as potent in activating the RAS pathway as V600 mutation in *BRAF* [15].

**Table 2:** Detailed assessment of the 24 CUP cases harboring a *BRAF* gene mutation

BRAF mutation	CUP histotype	Sex	EGFR status	Other mutations
V600E	Adenocarcinoma	F	Negative (FISH)	w.t. KRAS
V600E	Undifferentiated^1^	M	Negative (FISH)	w.t. KIT, KRAS, PIK3CA
V600E	Adenocarcinoma	F	Negative (FISH)	None (NGS panel)
V600E	Adenocarcinoma	F	Low (RT-PCR)	w.t. *KRAS, KIT, NRAS, ALK, PIK3CA*
V600E	Carcinoma NOS	F	Low (RT-PCR)	w.t. *KRAS, NRAS, PIK3CA*
V600E	Carcinoma NOS	M	Low (RT-PCR)	w.t. *KRAS, PIK3CA*
V600E	Adenocarcinoma	F	Positive (IHC)	None (NGS panel)
V600E	Adenocarcinoma	F	Positive (IHC)	*MET* T1010I, *TP53* R282W
V600E	Melanoma^2^	M	Negative (IHC)	w.t. *KRAS, PIK3CA, KIT, NRAS*
G464E^3^	Adenocarcinoma	F	Negative (IHC)	*KRAS* G12D
V600E	Neuroendocrine	M	Positive (IHC)	w.t. *KRAS, NRAS, PIK3CA, KIT*
V600E	Adenocarcinoma	F	n/e	None (NGS panel)
V600E	PDC	F	n/e	None (NGS panel)
G469A^3^	Adenocarcinoma	F	n/e	None (NGS panel)
D594G^4^	Adenocarcinoma	F	Positive (IHC)	*TP53* G108S, *KRAS* A146V; *APC* P1361L (VUS)
D594G^4^	Adenocarcinoma	F	Positive (IHC)	*TP53* E285K, E287D
G596R	Neuroendocrine	F	n/e	None (NGS panel)
D594N	Undifferentiated	F	Positive (IHC)	*KRAS* G13D
G464V	Adenocarcinoma	M	Positive (IHC)	*ERBB2* V777L; *STK* 11F345L
N581I	Squamous cell	M	Positive (IHC)	*GNAS* R201H; *ATM* V410A
N581S	Carcinoma	F	Negative (FISH)	None (NGS panel)
N581L	Carcinoma	M	EGFR M600T	None (NGS panel)
V600E	Adenocarcinoma	F	n/e	*MET* E168D (VUS); *PIK3CA* E545K
G466V	Undifferentiated	M	Positive (IHC)	*TP53* R273H

1 All cases of undifferentiated tumors, carcinoma not otherwise specified (NOS) or poorly differentiated carcinomas (PDC) were negative for melanocytic markers including S-100, HMB-45 and/or Melan-A.

2 Initial referring lab immunohistochemical analysis included negative S-100, positive vimentin and focal pan-keratins. Carcinoma was favored, but upon finding of BRAF V600E the tumor was further evaluated and found to be Melan-A+ and HMB-45+, consistent with malignant melanoma

3 Pathogenic, activating mutation

4 Presumed pathogenic mutations, sensitive to MEK inhibitors

n/e - not evaluable

PDC = Poorly differentiated carcinoma

w.t. = wild type

NGS panel included 47 genes; list available at: http://www.carismolecularintelligence.com/next-generation-sequencing-profile

VUS = Variant of Unknown Significance

NOS = Not otherwise specified

H-score was applied for EGFR protein estimation by immunohistochemistry (IHC); the score was obtained by the following formula: 3x% of strong membranous positivity + 2x% of moderate membranous positivity + 1x% of weak membranous positivity, giving a range of 0 to 300. Score ≥ 200 was considered positive.

Although molecular profiling of CUP has a limited potential in identifying the primary site of a tumor, in some cases it can lead to the refinement of the diagnosis. In a case submitted for analysis as melanoma from an unknown cutaneous primary site, metastatic to the T10 intradural space, our profiling identified *GNA11* Q209L mutation. This result identified the tumor as a primary leptomeningeal melanoma because of the characteristic association between the mutation and this tumor type [16].

## DISCUSSION

CUPs pose diagnostic and therapeutic challenges due to uncertain tissue of origin in the era of lineage-based (histology driven) therapies. However, recent work from The Cancer Genome Atlas project demonstrated that the tissue of origin of a cancer may be much less relevant to prognosis and response to therapy than identification of causative mutations [17]. These studies identified shared biomarkers, potentially responsive to drug therapies across a diverse group of cancers with known primary sites. We report here that a similar approach should be employed in cancers of unknown primary sites using comprehensive, multiplatform biomarker testing.

Sequencing of CUPs detected a limited variety of genetic mutations (only 5 genes were mutated at ≥5% frequency) with rather narrow therapeutic (actionable) applicability. Some of the detected mutations, albeit at low frequency, have not been previously reported in CUPs, including *cKIT* mutations, but may be of clinical relevance for the patients with refractory, metastatic disease, as exemplified by a case report where a patient with an activating *KIT* mutation and protein overexpression was successfully treated with sunitinib [14]. Similarly, CUP cases that harbored activating *EGFR* mutations were successfully targeted by EGFR inhibitors (e.g. gefitinib), as shown in our cohort and in two recent reports [18, 19]. Furthermore, a subset of CUP cases in our cohort harbored potentially actionable *HER2* and *EGFR* amplifications, which may benefit from multiple targeted treatment modalities [20, 21].

For the most part, results of our mutational analysis are comparable with the data published in two recent limited studies of CUPs [19, 22].

Our study showed that the vast majority of druggable targets in CUPs were identified using established protein biomarkers using standard, widely available IHC techniques. These biomarkers can be used to tailor therapeutic modality utilizing conventional therapies, in addition to targeted biological drugs in CUPs. For example, we profiled a case of poorly differentiated carcinoma metastatic to liver (CK+/CK7-/CK20-/TTF1-/p63+/SYN-/CD56-) who, after 6 cycles of carboplatin/paclitaxel had liver recurrence. Caris molecular profiling identified TOPO1+/Thymidylate synthase- immunoprofile of the tumor, indicating a potential therapeutic benefit from irinotecan/5-FU based chemotherapy. The patient was thus treated with FOLFIRI and at 6 months significant tumor reduction was seen (please see Acknowledgement section for cases described in the manuscript).

We also note the importance of interpretation of the biomarker expression within the context of relevant histologic classification of CUPs. For example, activating *BRAF* mutations (e.g. V600E) have been associated with a benefit using vemurafenib in melanomas, but not in colorectal carcinomas due to the activation of EGFR pathway in colon cancer [23]. As Table 2 indicates, we found *BRAF* activating mutations in 24 CUP cases, one of which was a melanoma without evidence for EGFR pathway alterations while more than half of the remaining twenty-three cases show potential EGFR pathway activation, suggesting they may not be ideal candidates for *BRAF* targeted mono-therapy. However, such cases may be considered for the combination therapy targeting both *EGFR* and *BRAF,* as had been described in a patient with *BRAF* V600E mutated colorectal cancer [24].

Alterations within the PI3K/AKT/mTOR signaling pathway are among the most frequent in human cancers [25–27]. Here, we describe frequent alterations of the pathway including PTEN protein loss and mutations, as well as *PIK3CA* mutations, which may provide a rationale for the targeted therapy with PI3K/AKT/mTOR inhibitors in up to 49% of CUPs.

Recently, PD-1 and PD-L1 have been identified as potential immune therapy biomarkers not just in melanomas but also in various solid malignancies including renal cell carcinoma and non-small cell lung carcinoma [28, 29]. Our study revealed the presence of these two immune check-point proteins in a subset of CUPs, which may open an unexplored avenue for the clinical trials with anti-PD1/PD-L1 antibodies in these patients.

This study has several limitations. Due to the low frequency of occurrence of CUP, a lack of prospective clinical trials exist by which to determine the true clinical significance of biomarkers tested in our patient cohort. Therefore, we relied primarily on the available evidence, which consisted primarily of single case reports. In the absence of robust clinical studies, case reports may be relevant for treatment of CUP [30]. Some of the biomarkers investigated in this study over a period of time have accumulated contradictory evidence on their predictive role (e.g. ERCC1) [31], which could be due to differences in analytic and interpretation platforms. Further, many confounding factors may contribute to patient response to therapy including gender, age, specific haplotypes, life style and comorbidities, which all were beyond the scope of this study.

In conclusion, pathway analysis and identification of biomarkers for targeted therapies is applicable to CUP and identifies many individualized treatment options which are not traditionally considered for CUP. Common molecular alterations identified in many CUPS indicate a potential paradigm shift in management of CUP by the inclusion of pathway driven therapeutic strategies, as supported by several recently published case reports [14, 18, 19].

## MATERIALS AND METHODS

### Case selection

A database search of cancer patients profiled at Caris Life Sciences (Phoenix, AZ) identified 1,806 consecutive CUP cases from more than 63,000 cases (approximately 3%). Multiple profiling of tumors from different sites or at different times in the same patients was performed in 103 of these cases (6%).

### Methods

The Caris Molecular Intelligence (CMI) tumor profiling service (available at: http://www.carismolecularintelligence.com/) is a CLIA/CAP/ISO certified laboratory which utilizes multiple standard platforms and methodologies, including tumor protein expression [immunohistochemistry (IHC)] (23 markers, Figure 2), somatic gene mutation analysis [Next-generation sequencing (NGS), Sanger sequencing, pyrosequencing, qPCR, and Restriction fragment length polymorphism (RFLP)]) analysis] (47 genes total, the list is available at: http://www.carismolecularintelligence.com/next-generation-sequencing-profile), and tumor gene copy number alterations [DNA in-situ hybridization: Fluorescent (FISH) and chromogenic (CISH)] (7 genes: *EGFR, HER2, TOP2A, cMET, PIK3CA, ALK, ROS1*), DNA fragment analysis and in some cases quantitative mRNA analysis (RT-qPCR) [32].

The associations between a biomarker result and drug benefit(s) were determined using recommendations from published clinical evidence in humans, which includes peer-reviewed literature and/or the National Comprehensive Cancer Network (NCCN) guidelines [33], not restricted to cancer type. No preclinical or experimental (unapproved) drug associations were made; however, biomarker associations to drugs in advanced stage clinical trials were made.
